# A decade-long silent ground subsidence hazard culminating in a metropolitan disaster in Maceió, Brazil

**DOI:** 10.1038/s41598-021-87033-0

**Published:** 2021-04-08

**Authors:** Magdalena Vassileva, Djamil Al-Halbouni, Mahdi Motagh, Thomas R. Walter, Torsten Dahm, Hans-Ulrich Wetzel

**Affiliations:** 1 GFZ German Research Centre for Geosciences, Telegrafenberg, 14473 Potsdam, Germany; 2 Institute of Photogrammetry and GeoInformation, Leibniz University Hannover, Nienburger Str. 1, 30167 Hannover, Germany; 3 Institute of Geosciences, University of Potsdam, Karl-Liebknecht-Str. 24-25, 14476 Potsdam-Golm, Germany; 4 Present Address: GEOMAR Helmholtz-Centre for Ocean Research, Wischhofstr. 1-3, 24148 Kiel, Germany

**Keywords:** Natural hazards, Solid Earth sciences

## Abstract

Ground subsidence caused by natural or anthropogenic processes affects major urban areas worldwide. Sinkhole formation and infrastructure fractures have intensified in the federal capital of Maceió (Alagoas, Brazil) since early 2018, forcing authorities to relocate affected residents and place buildings under demolition. In this study, we present a 16-year history (2004–2020) of surface displacement, which shows precursory deformations in 2004–2005, reaching a maximum cumulative subsidence of approximately 200 cm near the Mundaú Lagoon coast in November 2020. By integrating the displacement observations with numerical source modelling, we suggest that extensive subsidence can be primarily associated with the removal of localized, deep-seated material at the location and depth where salt is mined. We discuss the accelerating subsidence rates, influence of severe precipitation events on the aforementioned geological instability, and related hazards. This study suggests that feedback destabilization mechanisms may arise in evaporite systems due to anthropogenic activities, fostering enhanced and complex superficial ground deformation.

## Introduction

Land subsidence affects many highly populated urban areas of the world, either as a consequence of extensive groundwater depletion, such as in Tehran^1,2^, Las Vegas^3^, Beijing^4^, and Tucson^5^, as a combined effect of loading and compaction of unconsolidated lacustrine sediments, such as in Mexico City^6,7^, or via construction dewatering^8^ and underground mining^9–11^.

However, naturally or anthropogenically induced evaporite dissolution with consequent ground subsidence also occurs in several parts of the world, such as the salt dissolution cases of the Permian and Triassic evaporitic terrain in the UK^12^, numerous Triassic and Tertiary evaporite areas in Spain^13^, Quaternary sediment subrosion in the Dead Sea^14–16^ and many areas underlying the Permian basin in the United States^17,18^. Evaporite dissolution and consequent ground subsidence pose a severe geohazard for overlying urban areas, such as Zaragoza city in Spain^19^, Tuzla in Bosnia and Herzegovina^20^, and Wieliczka in Poland^21^.

In particular, salt (halite, or NaCl) is the most soluble evaporite rock that is widespread in continental regions. Freshwater percolation through halite layers rapidly dissolves these evaporites, leading to the formation of subsurface voids that, as they widen, can reach unstable conditions and provoke the roofs of these voids to collapse. A series of successive roof failures can cause the cavity to migrate upward, reaching the overburden layers. If the cavity’s roof, i.e., the rocks above it are not rigid enough, the cavity may collapse, with surface effects that can range from slow subsidence to sudden collapse and formation of sinkholes^18^.

**Figure 1:**
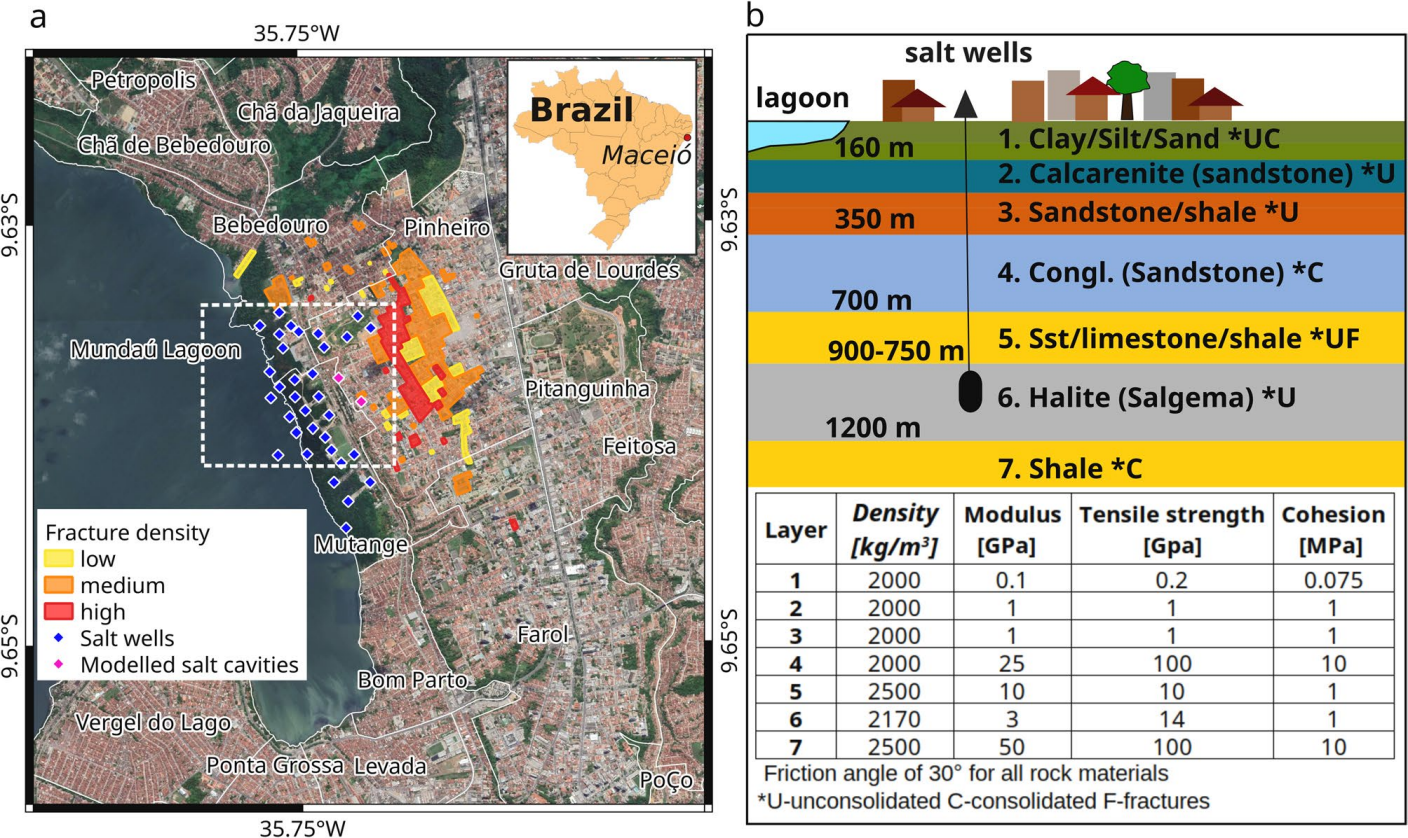
(**a**) Overview of the study area. Yellow, orange, and red polygons represent respectively areas with low, medium and high concentrations of fractures in buildings and infrastructures (assessment conducted by CPRM in 2018^25^). Blue and magenta diamonds show the locations of all installed salt wells since 1970. Specifically, magenta diamonds highlight the two cavities used in the distinct element method. The white dashed polygon is the area in Fig. 3a. Inset shows the geographical location of Maceió. (**b**) Simplified geological stratigraphic model and table of the rock material properties used in this study. Background in **(a)** Google Earth CNES/Airbus imagery. The map in **(a)** was plotted in QGIS (v 3.16, https://www.qgis.org/en/site/).

Solution mining refers to the extraction of salt by injecting water through wells drilled into subterranean deposits, dissolving the salts and pumping the resulting brine back to the surface, leaving brine-filled cavities behind^22,23^. Since 1970, a total of 35 industrial brine extraction wells have been installed along the Mundaú Lagoon coast in the urban area of Maceió, and more precisely, in the neighbourhoods of Mutange, Bebedouro, and Pinheiro (Fig. 1a). Maceió, the capital city of the Brazilian state of Alagoas, lies in the Sergipe-Alagoas salt basin, which formed along the Brazilian coast during South Atlantic rifting and was initiated in the Late Jurassic to Early Cretaceous. A variety of unconsolidated and consolidated sediments associated with different geneses and geological periods fill the basin.

At the beginning of 2018, fractures on both buildings and roads started to develop in the neighbourhood of Pinheiro following a rainfall event on the 15^th^ of February and a magnitude 2.4 earthquake (Brazilian local magnitude scale) on the 3^rd^ of March (Fig. 1a). Due to the high geohazard impact on the local population, the case received much national media attention. A total of 6,356 buildings were classified as risk zones and placed under demolition by the Brazilian authorities, with consequences for 25,000 residents, who were or still have to be relocated to other parts of the city, and considerable changes occurred for the urban setting of the affected districts^24^.

Several causes, including water depletion and pre-existing geological structure reactivation, have been investigated by the Brazilian Geological Service (Serviço Geologico do Brazil—CPRM), who performed a systematic survey and analysis between 06.2018 and 04.2019^25^. Recently installed seismic stations registered very shallow seismicity (hypocentre < 1 km) under the lagoon and the neighbourhood of Pinheiro on the 1^st^ of February 2019. A gravimetry survey showed negative anomalies (bodies with a lower density than the surrounding rocks) over the salt extraction area. An audio-magnetotelluric (AMT) investigation also detected low conductivity at approximately 900 m depth, which corresponds to the underground extraction layer. Sonar measurements of the salt cavities have detected upward migration and enlargement and occasional total or partial collapses in most of them. The 3rd of March 2018 seismic event with a hypocentre of approximately 1 km was later attributed to possible cavity collapse. Geodetic measurements using Sentinel-1 SAR data during 04.2016 and 12.2018 detected cumulative subsidence reaching 40 cm with a maximum close to the lagoon shoreline. Geological and geotechnical observations also identified several very shallow discontinuities visible in outcrops that have fostered erosion effects due to surface water infiltration, further increasing the geological instability.

In this study, we present a 16-year history of the spatio-temporal evolution of subsidence in the city of Maceió. For this purpose, we analysed a large archive of synthetic aperture radar (SAR) data from past and currently operational satellite missions between 2004 and 2020, highlighting the importance and effectiveness of the Interferometric SAR (InSAR) technique for monitoring geological instabilities. To test the underlying cause of the subsidence pattern, we used 3D geophysical source inversion and 2D geomechanical simulation. Different 3D elastic source models were tested to explain the overall deformation pattern. The distinct element method (DEM) allowed us to explicitly analyse subsidence due to mechanical failure of deep-seated cavities along a 2D transect in the regional geologic setting. We investigate the possible influence of meteorological factors and discuss whether the subsidence has been constant or accelerated in recent times. We further exploit interferometric measurements to highlight the dynamic evolution of the subsidence hazard by generating dynamic geohazard maps that are valuable for further infrastructure risk assessment.

## Results

### Spatio-temporal evolution of subsidence

**Figure 2:**
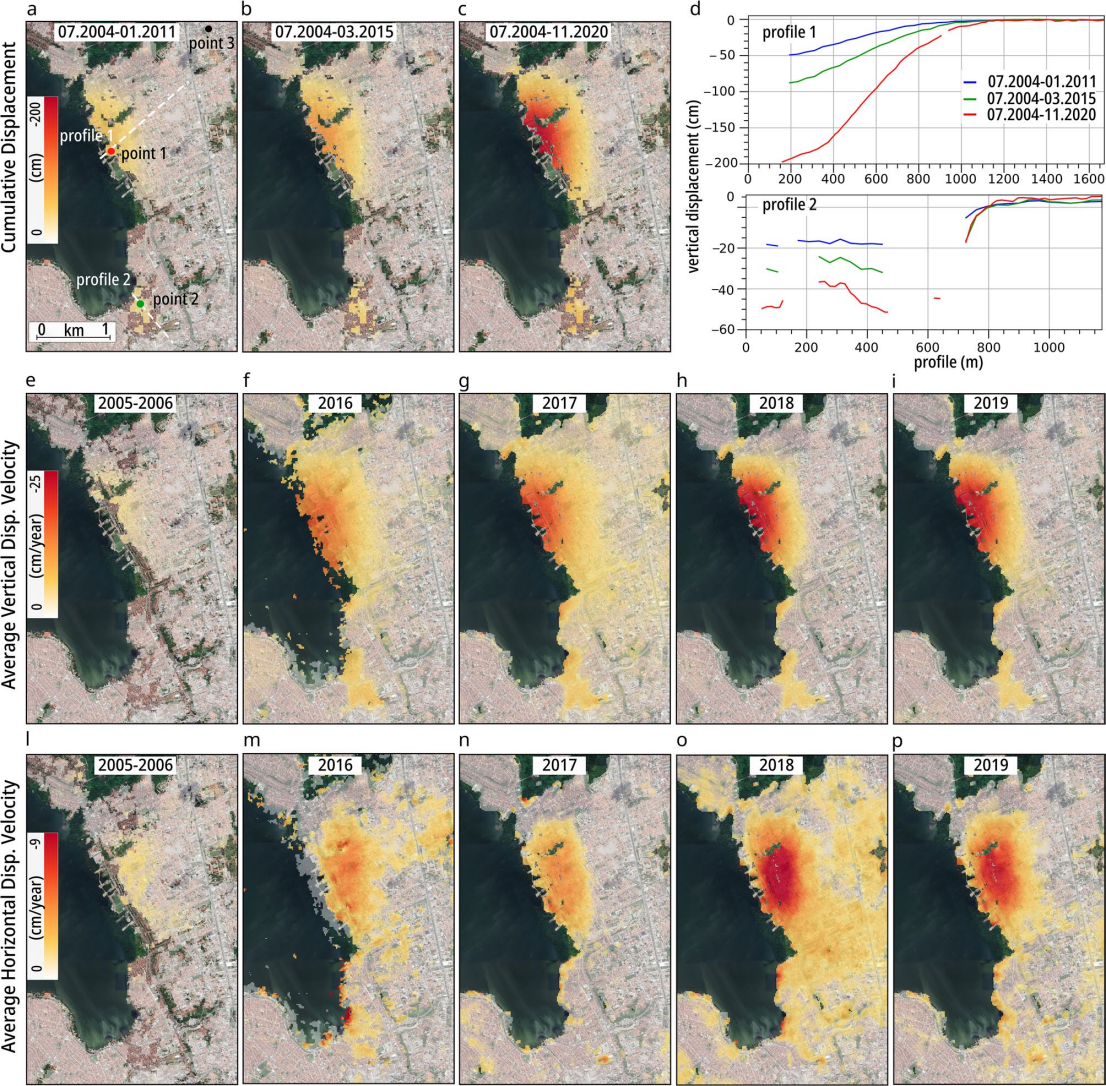
InSAR time series results. (**a–c**) Cumulative vertical subsidence maps obtained by projecting the LOS component into vertical only and combining in time and space all available displacement datasets. Red, green, and black points show the locations of the time series plotted in Fig. 6 respectively point 1 (in the main subsiding area), point 2 (in the minor subsiding area) and point 3 (in hypothetically stable area). White-lines show profile 1 and 2 plotted in (**d**) where the blue line refers to the period 07.2004–01.2011, green for 07.2004–03.2015, and red for 07.2004–11.2020. Ascending and descending displacements have been combined for the periods where both geometries were available to retrieve (**e–i**) vertical and (**l–p**) horizontal average displacement velocities. The horizontal negative values refer to westward motion. Background Google Earth CNES/Airbusimagery. The figures (except **d**) were plotted in QGIS (v. 3.16, https://www.qgis.org/en/site/).

Multi-temporal and multi-sensor InSAR processing (see the “Data and methods” section) have resulted in a high-resolution ground subsidence map of Maceió (Fig. 2). This map shows the spatio-temporal evolution of the subsiding area, which affects large parts of the neighbourhoods of Bebedauro, Mutange, and Pinheiro (Figs. 1 and 2). Early in the time series, since at least the second half of 2004, concentrically shaped subsidence patches gradually started to develop close to the Mundaú Lagoon coast with an initial maximum average velocity of approximately 4 cm/year (Figs. 2 and 6a). In the following years, the displacement gradually intensified to approximately 10 cm/year in 2007–2008 and reached approximately 12 cm/year in 2010–2011. In the second period of SAR data coverage that extends from 03.2015 to 11.2020, an initial subsidence velocity of approximately 12 cm/year was observed (2015–2016), which is similar to the period of 2010–2011. We assume that during the data gap from 02.2011 until 03.2015, the subsidence rate did not change. A slight increase in velocity to 17 cm/year was observed in 2016–2017, which then drastically increased during the second half of 2017, reaching a maximum of 27 cm/year (Fig. 6a,b). As the rate of subsidence has increased, the area affected by subsidence has also enlarged considerably. The maximum velocity has decreased to 20 cm/year since the beginning of 2020. A maximum cumulative ground subsidence of approximately 50 cm (over the six-and-a-half-year observation period of the first dataset), 46 cm (over the four-year data gap using data interpolation), and 105 cm (over the five-and-a-half-year observation period of the second dataset) was estimated for the three periods, with a total maximum subsidence for the whole period from 07.2004 until 11.2020 of more than 2 m (Fig. 2c,d).

For the periods where both ascending and descending SAR acquisitions were available, we also derived the east–west horizontal component of displacement (Fig. 2l–p)^26^. The horizontal displacement maps show a westward motion in accordance with the slope of the subsidence, which increases with increasing subsidence, although the displacement is still a few cm/year. The area of maximum horizontal displacement does not coincide with the area of maximum subsidence since the horizontal component is related to the vertical displacement gradient rather than its absolute value. Therefore, our projection of the line-of-sight InSAR displacement in the vertical direction is a valid approximation in areas where the subsidence reaches its maximum values.

From the area covered by InSAR observations, we estimated a minimum cumulative surface volume loss of 7.9E + 05 m^3^. However, the volume loss is much larger because a considerable part of the displacement is hidden underwater.

The vertical displacement time series also highlights other regions of ongoing subsidence. South of the lagoon, ~ 3 km south of the main subsidence region, we find localized subsidence that has been occurring since 2007, which affects parts of the coastal districts of Bom Parto and Levada. Subsidence in this location has been characterized by an almost constant average vertical velocity of 4 cm/year until the beginning of 2020, after which the trend has quite suddenly decreased to 1 cm/year and sometimes to 0 cm/year (Fig. 6a,b). While this trend differs from the accelerating trend in the main subsidence region, the vertical displacement map suggests that the two subsidence regions are spatially connected through a displacement pattern that can be traced along large parts of the coast and that is characterized by an NNW-SSE orientation, which is possibly indicative of a much larger source region (Fig. 2e–i).

### Modelling the subsidence cause and processes

Ground subsidence observations in urban areas can be better understood by simulating source processes, which we approached using two modelling strategies. First, we realized a source inversion that considered simplified sources in elastic host rocks only. Second, we develop more complex numerical models to explore the propagation of subsurface cavities, changing stress conditions, fracture formation, and subsidence.

The geodetic data inversion was derived for two displacement source models: point model^27^ and rectangular crack model^28^ in isotropic elastic half-space. For the point model, the observed ground subsidence is assumed to be related to a sub-ground pressure change caused by a spherical depressurised point source. This is then converted to volume. For the rectangular crack model, the observed subsidence is assumed to be related to a volume change due to a near-horizontal fracture that is closing. Both source models can be associated with the withdrawal of fluids and/or removal of sub-ground solid materials^29,30^. In the case of salt mining, the volume loss might be attributed to the extraction of salt^31^. The search for the best modelling parameters was performed in a non-linear inversion scheme (see “Data and methods”) by repeating hundreds of simulations, until the misfit between the data and model is minimized. The resulting point pressure model provides a good approximation of the centre of the displacement source, while the rectangular crack model allows the retrieval of information regarding the possible spatial distribution and orientation of the displacement source.

**Figure 3:**
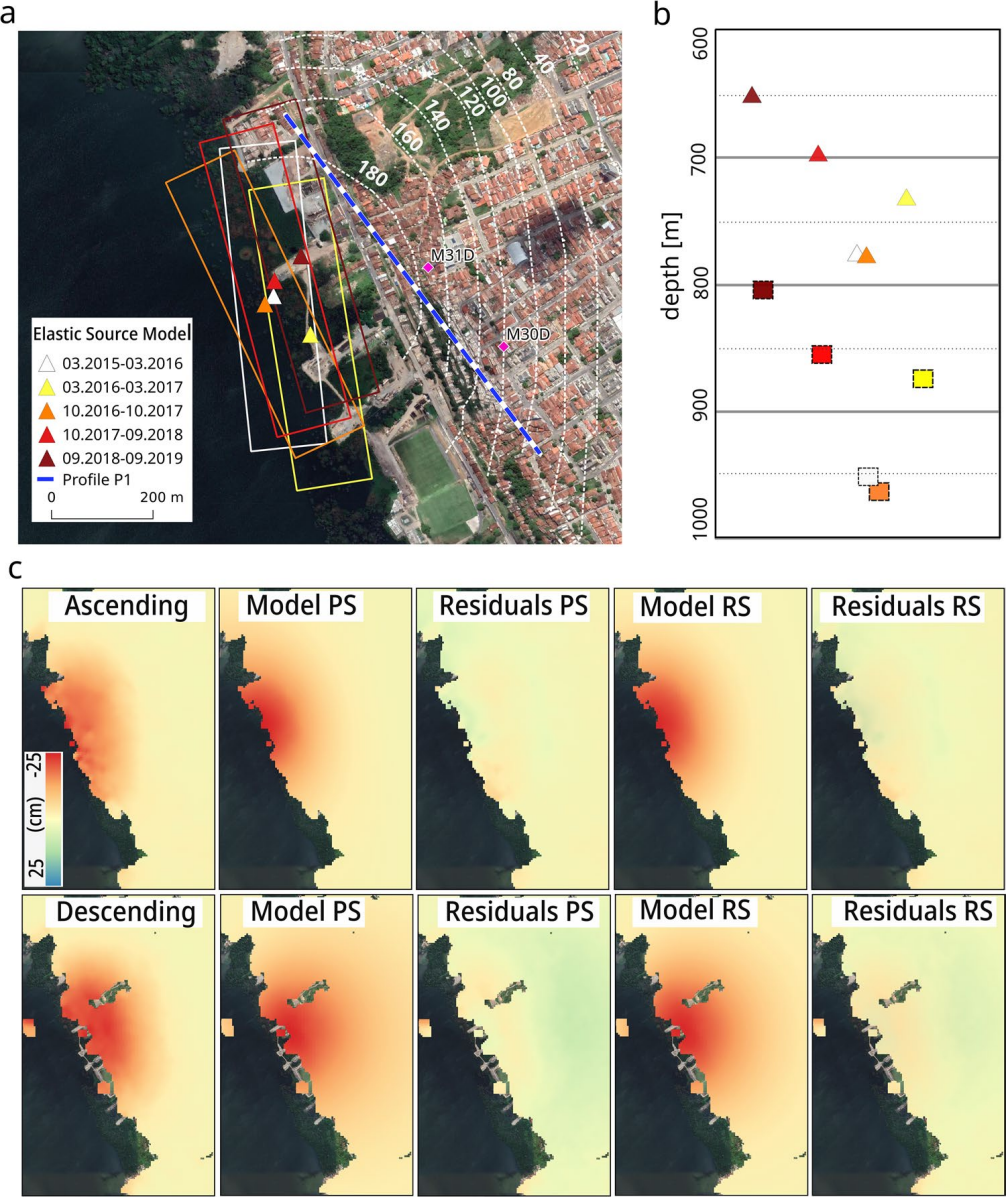
Inverse numerical model results. (**a**) Horizontal location of the best-fitting source models between 2016 and 2019: triangle symbology for point source model and solid rectangle for rectangular crack source model with different colours expressing the different dates as in legend. Dashed white isolines represent the cumulative displacement for the period 07.2004–11.2020. Magenta diamonds show the detailed location of the two cavities (M31D and M30D) modelled in DEM. The blue-white line shows the profile P1 used for the DEM subsidence simulation (see Figs. 4b and 5b). **(b)** Vertical profile reveals the depth of the best-fitting source models: triangles for point pressure source and rectangles for rectangle crack source (same colour convention indicating the date). X-axes is an indicative NW–SE along with the coast profile, not in scale. **(c)** InSAR ascending (Alos-2 data) and descending (Sentinel-1 data) observations for the period 2018–2019, best-fit model and relative residuals calculated by subtracting the model from the observations. PS indicates point source model; RS indicates rectangle crack source model. Background Google Earth CNES/Airbus imagery. The figures (except **b**) were plotted in QGIS (v. 3.16, https://www.qgis.org/en/site/).

The retrieved point pressure and rectangular crack source parameters for five different one-year intervals are shown in Table 1 (Fig. 3a,b). The best-fitting source models (Fig. 3c) are located at a depth 600–1000 m, which is coincident with the halite layer (750–950 m). In the point pressure model, the centre of displacement, and therefore the horizontal source location, remains constant through time and coincides with the centre of the salt mining area, while the rectangular crack model shows a SE-NW source orientation, which is in alignment with the spatial distribution of the wells. A general upward movement is visible from the two models: from 774 to 653 m for the point pressure source model and from 953 to 807 m for the rectangle source model. A comparison of the two models shows that the point pressure source model results in higher volume changes though a shallower source depth compared to the rectangular crack model. A volume loss on the order of E + 05 m^3^, which is comparable to the size of a single salt cavity, occurs every year. Therefore, the hypothesis of salt dissolution as main causes of subsidence is plausible. A rapid increase in volume loss from 3.6E + 05 to 5.3E + 05 for the point pressure source and from 2.7E + 05 to 4.2E + 05 for the rectangular crack source appears between the second (03.2016–03.2017) and third (10.2016–10.2017) datasets and is accompanied by a downward movement of the source. These two datasets have a 5-month overlapping period, and therefore, the drastic volume change increase most likely occurred during the second half of 2017, which is coincident with the rapid displacement acceleration observed in the InSAR time series (see the “Discussion” section).

**Table 1:** Elastic modelling parameters for point source model and 600 × 150 m rectangular crack source model for five-time intervals.

Time interval	Point source model				Rectangular source model 600 × 150 m			
	East (m)	North (m)	Vol. loss (m^3^)	Depth (m)	Opening (m)	Vol. loss (m^3^)	Strike (°)	Depth (m)
03.2015–03.2016	198,124	8,933,762	3.9E + 05	774	− 3.4	3.0E + 05	175	953
03.2016–03.2017	198,198	8,933,687	3.6E + 05	730	− 3.0	2.7E + 05	171	873
10.2016–10.2017	198,108	8,933,746	5.3E + 05	777	− 4.6	4.2E + 05	155	962
10.2017–09.2018	198,127	8,933,793	5.8E + 05	697	− 5.2	4.6E + 05	165	857
09.2018–09.2019	198,179	8,933,841	5.4E + 05	653	− 4.9	4.4E + 05	164	807

More complex numerical models explore how such cavity sources may eventually develop into anelastic processes and subsidence^32^. Geomechanical models of the subsidence process have been developed to compare the InSAR subsidence along a 2D transect crossing the surface projection of salt cavities M30 and M31 (Figs. 1a and 3a), which are located inside the residential area of Pinheiro. Two independent injection pressure scenarios (S1 and S2) were used to test the different geomechanical stages of the cavity evolution, surrounding crack propagation, stress development, and induced surface displacement that occur under different initial conditions. The first scenario (S1) considers a higher cavity pressure compared to the surrounding soil and simulates mining conditions. The working pressure usually stabilizes the salt cavities during dissolution mining. The second scenario (S2) considers a hypothetical lower cavity pressure, caused by depressurization and aim to simulate inactive mining conditions ^25^.

**Figure 4:**
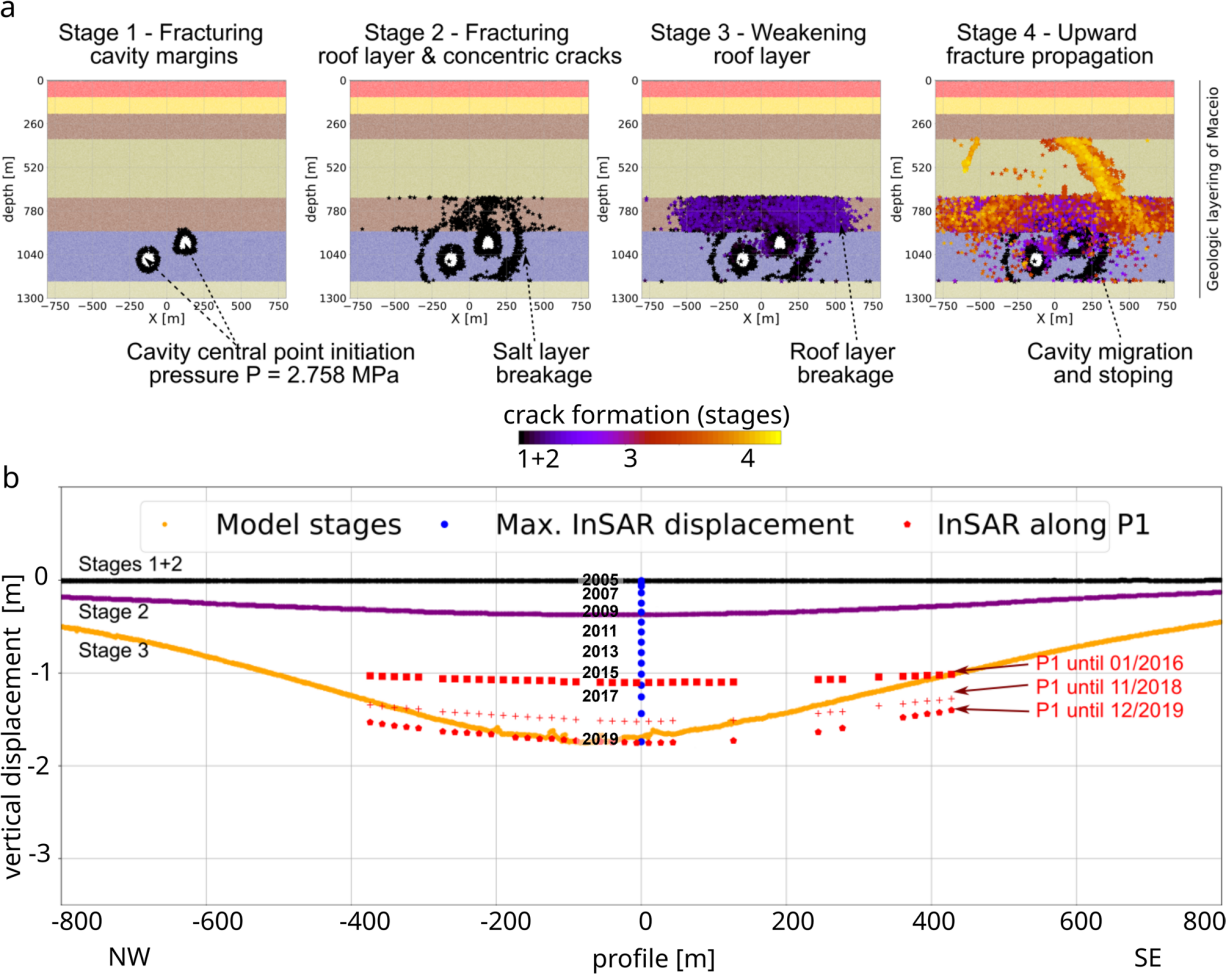
Simulated DEM subsidence models for pressurized cavity scenario (S1, P = 2.758 MPa). (**a**) Crack and fracture evolution representation of the four stages of cavity collapse shown in black–purple–yellow colour scale; the stratified background represents the geological layering model (see “Data and methods”). (**b**) Induced surface deformations for the four stages (black–purple–yellow colour scale) compared to InSAR surface subsidence results along the profile P1 (Fig. 3a). The figures were plotted using Matplotlib python library using data from simulations and InSAR time series data along profile P1.

From the first simulation scenario (S1) under working pressure conditions of P = 2.758 MPa, the following four model stages occur: (1) initial fracturing of the cavity margin due to the injected pressure; (2) fracturing of the roof layer, the formation of concentric cracks in the salt-rock layer around the cavities, and fracture propagation in the overburden shale layer; (3) weakening of the roof layer and collapse of the shallower cavity (M30); and (4) upward fracture propagation, cavity migration, and stoping. Only one cavity collapsed entirely under these pressure conditions. The crack evolution and simulated surface displacement compared to the subsidence InSAR observations are depicted in Fig. 4. The maximum subsidence reached at the final stage is 1.7 m, which occurs approximately 80 m NW of the central point of the profile and coincides with the maximum cumulative subsidence detected in that location in 10.2019. This final surface subsidence profile is rather smooth with little inhomogeneity due to discrete rock mass movement.

**Figure 5:**
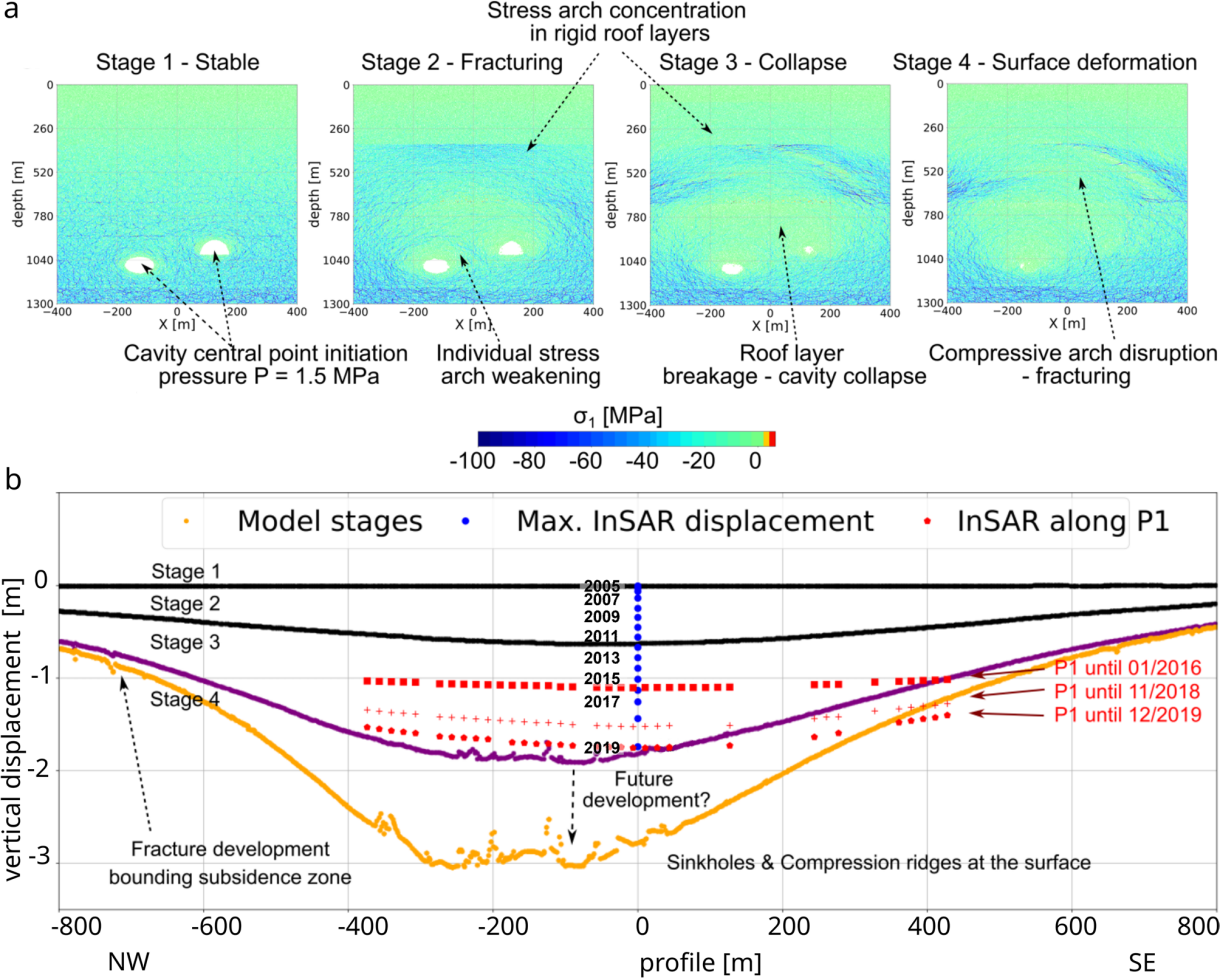
Simulated DEM subsidence models for depressurized cavity scenario (S2, P = 1.5 MPa). (**a**) Maximum compressive stress representation of the four stages of cavity collapse; blue colour shows higher values of compressive stress. (**b**) Induced surface deformations for the four stages (black-purple-yellow colour scale) compared to InSAR surface subsidence results along the profile P1 (Fig. 3a). The figures were plotted using Matplotlib python library using data from simulations and InSAR time series data along profile P1.

From the second, independent simulation scenario (S2) under inactive, depressurized initial conditions of P = 1.5 MPa, the following four model stages occur: (1) an initially stable pressurized cavity; (2) weakening of the individual compressive stress arches around the cavities and stress concentration in the large spanning compressive stress arch; (3) weakening of the roof layer, total collapse of the shallower cavity (M30) and partial collapse of the deeper cavity (M31), and fracture propagation into the overlying limestone/sandstone and shale (layer no. 5); (4) disruption of the large compressive stress arch, total collapse of the second cavity (M31) and upward fracture propagation with surface deformation. Crack evolution follows a similar pattern as the pattern in scenario S1 with working pressure conditions, although stages 3 and 4 are reached faster. Figure 5 shows the compressive stress conditions and the simulated surface displacement compared to the subsidence InSAR observations. After stage 3, a total maximum vertical displacement of almost 2 m was achieved at approximately 125 m NW of the central point of the profile, above the centre of cavity M31. Further, one metre of subsidence is related to the final stage 4, which indicates ongoing subsidence due to progressive collapses and compaction. This resulting final surface subsidence profile shows many inhomogeneities due to the development of fracturing and compression ridges at the surface.

Both simulated pressure cases show that the cavities already experience mechanical instability during working pressure conditions, with consequent roof collapses, upward cavity migration, and fracture propagation into rigid upper layers. The geomechanical condition is aggravated if the cavities were depressurized, leading to further collapses and ground displacement with more inhomogeneities at the surface.

## Discussion

In this study, we investigated the ongoing geological instabilities in Maceió by integrating multi-temporal InSAR analysis with source modelling using elastic inversion and the distinct element method. The main outcomes from our results are that the subsidence in Maceió (1) started to gradually evolve almost two decades ago with slow acceleration at the beginning and faster acceleration in the last 4 years; (2) reached a maximum cumulative value close to the lagoon coast of approximately 2 m at the end of 2020; (3) is attributable to a depth source between 600 to 1000 m that coincides with the salt cavity locations; (4) both active/pressurized and inactive/depressurized salt mining conditions led to mechanical instability of the cavities with local upward migration and likely partial to total cavity collapses; and (5) developed from the deforming cavities cracks propagated upward towards the shallower layers.

Almost two decades of displacement observations highlight the gradual spatio-temporal evolution of the main subsidence process. The displacement observations also show the presence of a second minor unstable area on the south coast of the lagoon that is characterized by block caving subsidence.

By integrating the displacement observations with numerical source modelling, we suggest that extensive subsidence can be primarily associated with the removal of localized, deep-seated material at the location and depth where salt is mined. This makes other explanations that associate geological instability with distributed surface water percolation or only with the destabilization of pre-existing geological structures highly unlikely. The DEM also shows that deep cavities in the “salgema” salt layer can, even under higher working pressure conditions, mechanically create cracks in the upper layers that eventually lead to large-scale subsidence and small-scale surface features. In conclusion, the deep mining horizon with resulting high surrounding environmental pressure and local rock mechanical conditions are the main reasons for the instability of cavities in this salt layer.

Rock fracturing, including in upper layers, as observed in Maceió, is an explicit indicator of geomechanical degradation. Cracking of the surface layers and weakening of the bulk material eventually enables strong water percolation from rather superficial aquifers into deeper underground areas, with a potential increase in material dissolution and erosion. This process can lead to a feedback mechanism responsible for superficial ground deformation and even to enhanced local subsidence. The connection between accelerated subsidence and extreme rainfall is further discussed. The ongoing process of mechanical destabilization is indicated by the fact that even though all mining activities have stopped since mid-2019, the displacement observations show a decreasing trend only from the beginning of 2020. Additionally, the known existing geological structures can foster water percolation and be reactivated if they spatially intersect the upward-moving cavities, provoking further surface displacement. This may be the genesis of the minor area of subsidence south of the lagoon, which subsides as a unique block, has an approximately constant rate and follows an NNW-SSE orientation, similar to the dominant regional fault system.

**Figure 6:**
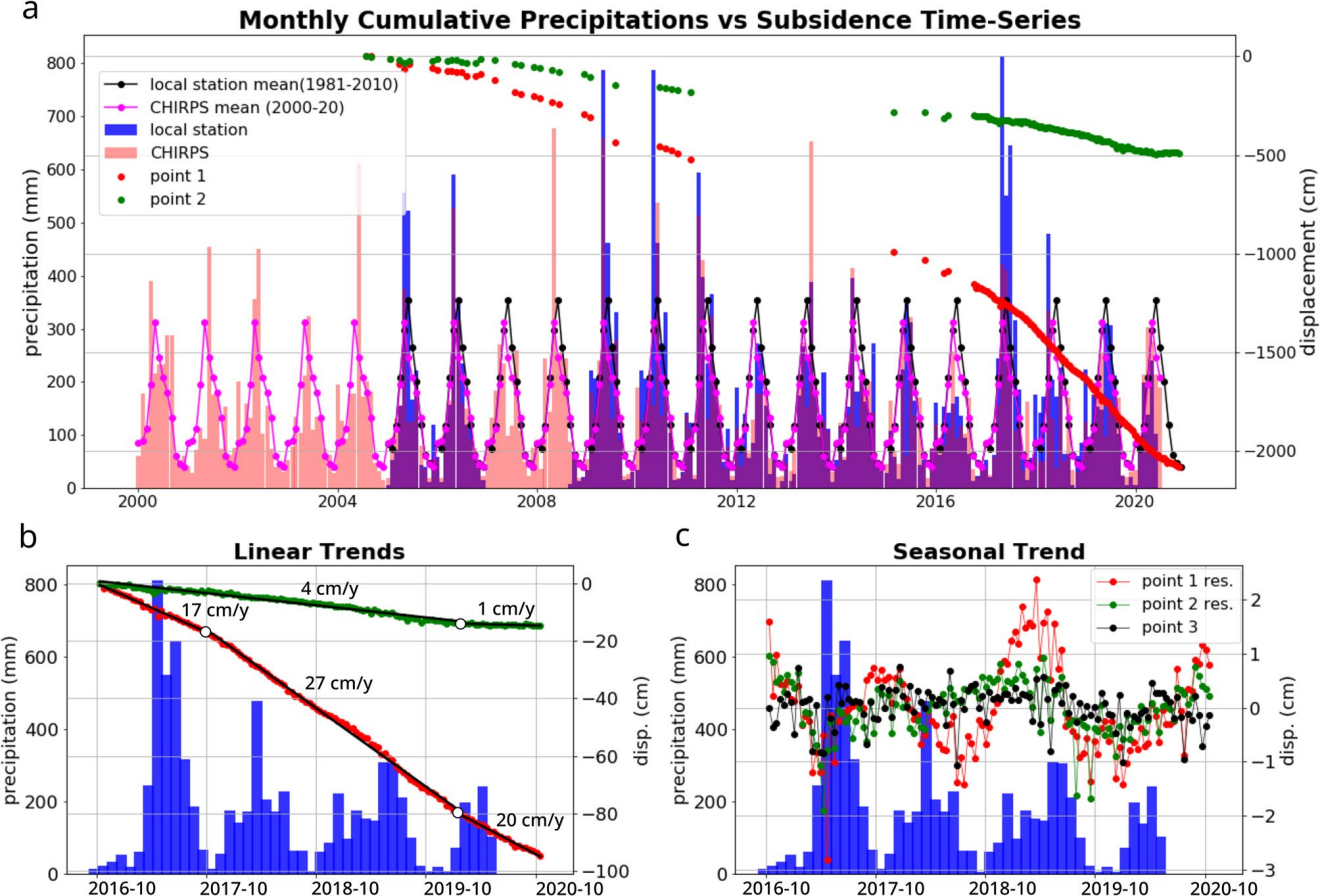
Long and short term vertical displacement time-series and local rainfall data. The location of the plotted points is shown in Fig. 2a. (**a**) 16-year long time-series: red (point 1) in the area of maximum displacement and green (point 2) in the minor subsiding region. Displacement values are on the right axis. Rainfall values are on the left axis: black dots and line for local station mean values; purple dots and lines for CHIRPS mean values; blue histogram for local station monthly cumulative precipitations; pink histogram for CHIRPS monthly cumulative precipitations. (**b**) 4-year short time-series for point 1 and 2 and histogram of local station monthly cumulative precipitations. Black-lines are the linear displacement interpolation representing the velocity trends; white dots shows changes in linear trend. (**c**) Seasonal trends: red and green dots of the residual estimated by subtracting a 3-grad polynomial trend from the displacement time-series for point 1 (point 1 res.) and point 2 (point 2 res.) respectively and black dots showing the displacement time-seires of point 3 in a potentially stable area. The figures were plotted using Matplotlib python library using InSAR time series and precipitation data.

In the 16-year long term, the InSAR data suggest a significant increase in subsidence rates. We examine the short-term and long-term fluctuations observed and compare them to extrinsic influences (Fig. 6). Specifically, our InSAR data suggest an acceleration in the subsidence rates in 2017 (Fig. 6a,b). This concurs with hydrometeorological extremes affecting the region. Precipitation data from the Maceió meteorological station integrated with the Climate Hazards Group InfraRed Precipitation with Station (CHIRPS) precipitation data suggest that the period of May–July 2017 was characterized by almost double the average rate of rainfall. Concurring with this rainfall event, the InSAR data show an acceleration of 10 cm/year during the second half of 2017 (Fig. 6b). More short-term fluctuations associated with annual rain are depicted, implying that rainfall control might only be relevant for rainfall cumulative extremes (such as in 2017) but not for season-dependent fluctuations (Fig. 6c). These observations may allow the development of a threshold for the trigger ability in the future but necessitates further studies on longer time series. Due to the low temporal resolution of the other SAR acquisitions in the period from 2004 to 2011, it is not possible to reliably identify any correlation between ground subsidence trends and precipitation.

Knowing the dimensions and changes of subterranean cavities is of major importance for engineering mining and hazard assessment. We herein compared the overall volume loss derived by the analytical model with the salt cavity sizes, to obtain an overall idea of the possible cavity collapses. To calculate the whole volume loss, we used forward modelling method to simulate the complete subsidence ellipsoid for the period of 2004–2020; we obtained a minimum overall volume loss of 26.6E + 05 m^3^, which is three times more than the volume loss estimated only from the InSAR observations. Considering an average salt cavity size of approximately 3E + 05 m3, the above volume loss is equivalent to the total collapse of almost nine salt cavities. This estimation has to be considered conservative (a “minimum”), as natural effects such as material dilation of the sediment cover and anthropogenic refilling of cavities have not been taken into account. Indeed, from elastic modelling, we obtain an approximate subsurface volume loss of 22.5E + 05 m^3^ for the point pressure source and of 17.7E + 05 m^3^ for the rectangular crack only for the 03.2015–09.2020 period.

In subsiding areas, the damage to buildings and infrastructures is related to the strain changes that occur due to differential settlement^33^. A good indicator of such a strain factor is the angular distortion, which is calculated as the ratio of the subsidence horizontal gradient, i.e., the differential settlement and the distance between the two considered points. Therefore, for infrastructure risk assessment and emergency management, angular distortion provides more appropriate information than displacement information alone. Moreover, since subsidence is a dynamic process, hazard evolution is dynamic.

**Figure 7:**
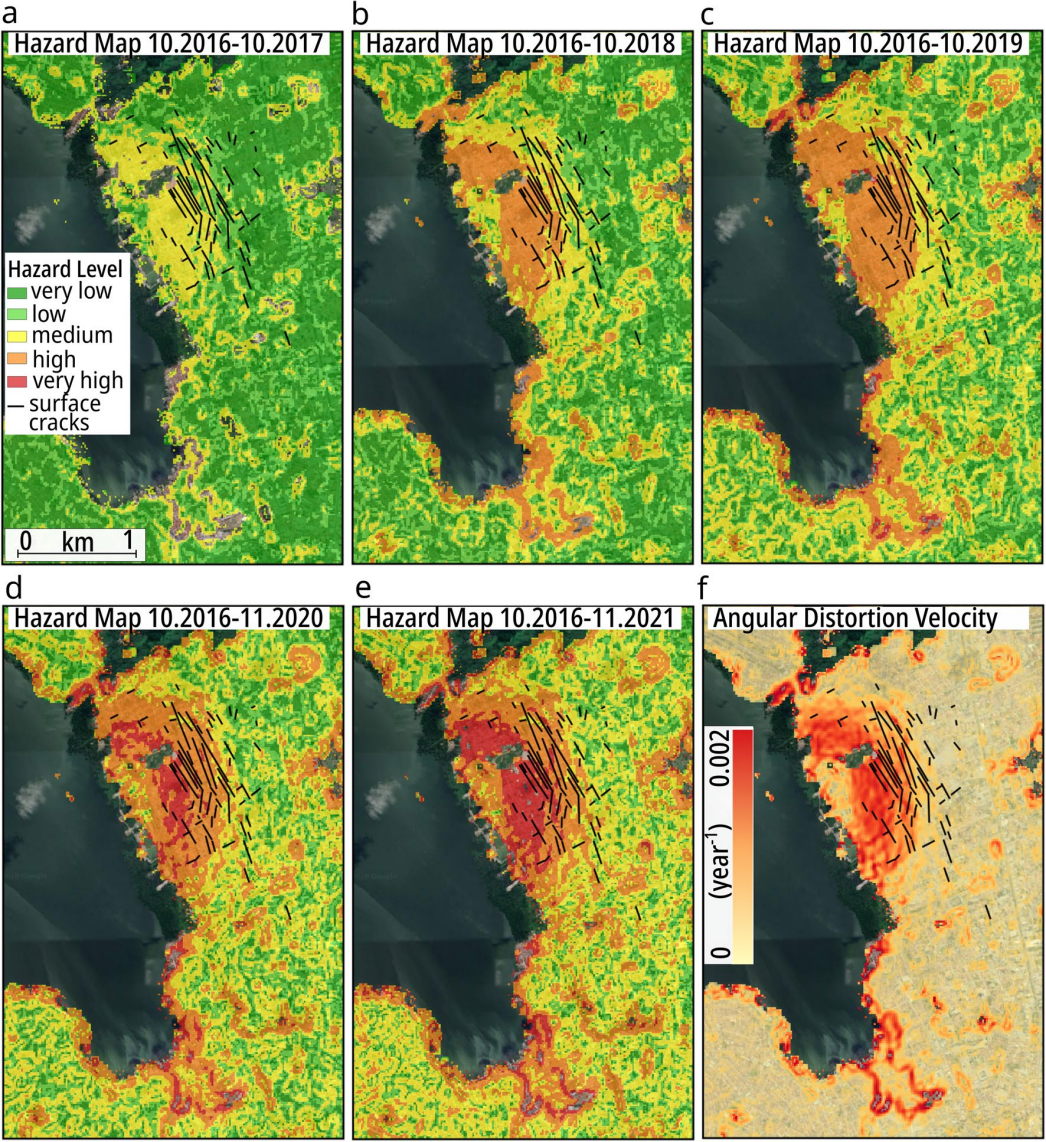
Subsidence hazard based on angular distortion values (horizontal strain). (**a–d**) Cumulative hazard maps are classified into five levels based on an appropriate threshold (see “Data and methods”). (**e**) Hazard simulation of accumulated subsidence predicted by adding one further year (11.2020–11.2021) assuming constant displacement rate same as 2019–2020 (**f**) Angular distortion average velocity, estimated over the period 2016–2020: red colour indicates areas with faster hazard evolution. Background Google Earth CNES/Airbus imagery. The figures were plotted in QGIS (v. 3.16, https://www.qgis.org/en/site/).

Based on the aforementioned assumption, we properly classified angular distortion into hazard levels (see “Data and methods”), and we derived cumulative geohazard maps for the last 4 years (Fig. 7a–d).

The relationship between high angular distortion and damage occurs in the zone where the ground gradually transitions from stable to unstable conditions. Indeed, the surface cracks detected during a ground survey conducted by the CPRM in 2018 occur in the region of higher angular distortion and form concentric patterns around the maximum subsidence area. The second area of subsidence south of the lagoon has higher hazard levels around the perimeter, which highlights block-wise subsidence. We estimated the cumulative subsidence hazard by simulating an additional year of subsidence at the same rate as that in 2019–2020 (Fig. 7e). Potentially high levels of future hazards may develop in the middle region of the concentrically shaped subsidence and then gradually develop towards both the east, i.e., the transition region, and west, i.e., the area of maximum subsidence. The dynamic character of the subsidence hazard is well depicted by the angular distortion average velocity map (Fig. 7f), where a higher velocity indicates the areas where the hazard evolves more rapidly.

In addition, some inland areas are also classified as having a high hazard level, though they are far from the main unstable region and include some edge effects along the lagoon coast. These areas must be separately investigated because they could either be related to local processes or be the product of InSAR processing errors, as discussed in the “Data and methods” section.

## Data and methods

### Multi-temporal DInSAR

We measured surface displacement for the last 16 years using the multi-temporal DInSAR technique and exploited the full archive of multi-sensor SAR data from past and currently operational satellite missions. We adopted the Small BAseline Subset (SBAS) algorithm^34^ implemented in the commercial software ENVI/SARscape. SBAS is based on a combination of interferograms characterized by small normal and temporal baselines, allowing us to maximize spatial and temporal coherence. The main characteristics of the six independent SAR datasets processed in this study are illustrated in supplementary Fig. S1 and Table S1. The Envisat ASAR C-band and the Alos-1 Palsar L-band SAR missions cover the period of 10.2003–01.2011. A four-year gap in acquisitions is present between 01.2011 and 02.2015. The currently operational Sentinel-1 C-band and Alos-2 Palsar L-band missions cover the period from 02.2015 to 11.2020. The SBAS connection graphs are plotted in supplementary Fig. S2. Some selected wrapped phase displacement maps are shown in supplementary Fig. S3.

Since both ascending and descending acquisitions covering the same period and with the same time resolution were available only for the periods of 03.2005–03.2006 and 03.2015–09.2019, we ignored the horizontal component and converted the line-of-sight (LOS), i.e., direction from the satellite to the ground, displacement into vertical-only components. For the data overlap periods, we chose the dataset characterized by higher spatial coherence and temporal density, while for the data gap period, we performed a polynomial regression considering the average velocities of one year before and one after the time gap.

We estimated the residuals between the decomposed vertical component and the simplified vertical-only component for the period of 10.2016–09.2019. The approximate error is equal to two-thirds of the horizontal velocity, and in the case of the westward horizontal component, the vertical displacement is overestimated in the ascending geometry and underestimated in the descending geometry when assuming a vertical-only component. Nevertheless, the final error in the area of maximum subsidence due to the vertical-only simplification for the Sentinel-1 dataset, which has a descending geometry, is on the order of 1–2 cm/year.

The SBAS overall velocity error was estimated for each dataset by calculating the velocity mean and standard deviation over regions assumed to be stable (supplementary Table S1). The estimated overall error is on the order of 1–2 mm/year in the LOS direction, which means for the cumulative displacement for the whole data period, the error is on the order of few cm. However, while this overall trend does not significantly affect the final interpretation and results, localized errors characterized by higher values may be present in the dataset, with consequent misinterpretations, i.e., a subsidence hazard in the region outside the main deforming areas.

### Inverse numerical modelling

We performed geophysical elastic source inversion using the modelling module of ENVI/SARscape and by jointly inverting one ascending and one descending measurement for five separate periods: 03.2015–03.2016 (Alos-2 PALSAR ascending and descending); 03.2016–03.2017 (Alos-2 PALSAR ascending and descending); 10.2016–10.2017 (Alos-2 PALSAR ascending and Sentinel-1 descending); 10.2017–09.2018 (Alos-2 PALSAR ascending and Sentinel-1 descending); and 09.2018–09.2019 (Alos-2 PALSAR ascending and Sentinel-1 descending). We constrained the source parameters by minimizing the misfit between predicted and observed surface displacements^35^. First, we subsampled the displacement datasets using a regular grid with two different sampling densities of 50 m and 150 m over the area of subsidence and the surroundings and generated a set of approximately 650 point measurements. Initially, we set up a point pressure source^27^ by leaving all source parameters unconstrained: volume change, depth, and coordinates of the source centre. Afterwards, we inverted the measurements for a rectangular model dislocation^28^ by assuming a pure vertical opening (dip = 0°, rake = 0° and slip = 0) and by fixing the horizontal location of the centre of the rectangle with the coordinates retrieved from the point pressure source modelling. We retrieved the best-fitting depth, strike, and opening values assuming a rectangular crack of 600 × 150 m. By varying the length and width parameters, the opening value changed; however, the volume change estimated as length*width*opening remained quite constant.

### 2D distinct element modelling

We performed 2D distinct element modelling (DEM) with PFC2D V5 software from Itasca. The DEM simulates the material as an assemblage of discrete and rigid particles of different radii and geomechanical parameters^36^. The particles are bonded together using the so-called soft-contact approach, which allows them to rotate and overlap at contact points, simulating mechanical interaction. For a proper representation of the matrix between grains, the parallel-bond scheme was used, which allows the simulation of shear and tensile crack formation and block rotations^37^. Based on the available stratigraphic information, we set up the configuration of the material layers and properties (supplementary Fig. S4). Detailed parameters of the simulated geologic materials and parameters as well as geometries are given in supplementary Tables S2 and S3. We installed the two cavities by deleting the particles at a specific depth and according to the size detected by the sonar measurements. The two cavities had centre point depths of 1010 m and 1070 m and sizes of approximately 14,100 m^3^ and 31,400 m^3^ for M30 and M31, respectively. Instantaneous particle deletion was followed by the setting of temporarily high bond strengths to avoid dynamic effects. To simulate the geomechanical behaviour of the subsurface, two independent scenarios, pressurized conditions (S1) and depressurized conditions (S2), were used. For S1, a pressure of 2.758 MPa, equivalent to the reported salt-mining pressure in this area, was initially injected into the cavity walls, which was simulated as explicit radial forces onto the inner rim particles. The modelling was redone with different initial conditions, including a lower pressure of 1.5 MPa and reproducing possible mining depressurized/inactive conditions. Different aspects should be considered in terms of uncertainty. First, it is important to highlight that the geomechanical model that was performed is a 2D model along a transect. The disk-shaped particles contain a third particle dimension of size one, which is added for correct calculations. Therefore, it might overestimate the instability due to missing bonds in the third dimension, and it is not possible to compare the volume changes directly with those retrieved in the 3D geophysical source inversion. Second, the model resolution (model size vs. particle radii) and bulk rock parameter calibration contain another uncertainty in particle-based simulations^37^. However, extensive experience with similar simulation setups has recently been achieved, and particle scale parameters have been adjusted by applying findings from available simulated compression and tension tests^32,38^ on material samples used in this study (consolidated rock, unconsolidated rock, and halite).

Third, DEM models have an intrinsic uncertainty due to random particle packing, a feature also observable in natural geologic depositional environments. Therefore, a repetition of four models per scenario was performed with different random particle assemblies. The resulting error margin in the subsidence calculation for the total collapse of both cavities (scenario S2) is plotted in supplementary Fig. S4b. The error is low at the margins of the 2D transect and higher with values up to approximately 50 cm in the part most affected by deformation. Due to the discontinuous nature of the model, each random assembly produces also different structures in the subsurface and at the surface. An even larger number of model generations would decrease the error. We restricted the detailed stress and crack analysis to a representative model for each scenario and have shown that the subsidence determined by InSAR is within the range of the simulated subsidence, even close to the mean of all assemblies.

### Geohazard maps

Geological instability hazard maps were produced based on the angular distortion^33,39,40^, which was calculated as the ratio of the subsidence horizontal gradient between two adjacent pixels to the horizontal distance between them, equivalent to 15 m of pixel size. The subsidence horizontal gradient was calculated from the Sentinel-1 LOS displacement maps for the cumulative periods of 10.2016–10.2017, 10.2016–10.2018, 10.2016–10.2019, and 10.2016–07.2020. We classified angular distortion into five hazard levels (supplementary Table S4) based on the limiting criteria available in the geotechnical literature and standards^33,41,42^.

## Supplementary Information

Supplementary Information.
