# Supplementary material for: A decade-long silent ground subsidence hazard culminating in a metropolitan disaster in Maceió, Brazil
Source: Sci Rep. 2021 Apr 8;11:7704. doi: 10.1038/s41598-021-87033-0 (PMC8032792; doi:10.1038/s41598-021-87033-0)
Supplement: Supplementary file 1 — Supplementary Information. [file 41598_2021_87033_MOESM1_ESM.docx]

A decade-long silent ground subsidence hazard culminating in a metropolitan disaster in Maceió, Brazil

**Magdalena Vassileva*^1,2^*, Djamil Al-Halbouni*^1*^*, Mahdi Motagh*^1,2^*, Thomas R. Walter*^1^*, Torsten Dahm*^1,3^,  *Hans-Ulrich Wetzel^1^*

^1^GFZ German Research Centre for Geosciences, Telegrafenberg, 14473 Potsdam Germany, e-mail magda88@gfz-potsdam.de

^2^Leibniz University Hannover, Institute of Photogrammetry and GeoInformation, Nienburger Str. 1, 30167 Hannover, Germany

^*^Now at: GEOMAR Helmholtz-Centre for Ocean Research, Wischhofstr. 1-3, 24148 Kiel, Germany

^3^University of Potsdam, Institute of Geosciences, Karl-Liebknecht-Str. 24-25, 14476 Potsdam-Golm, Germany


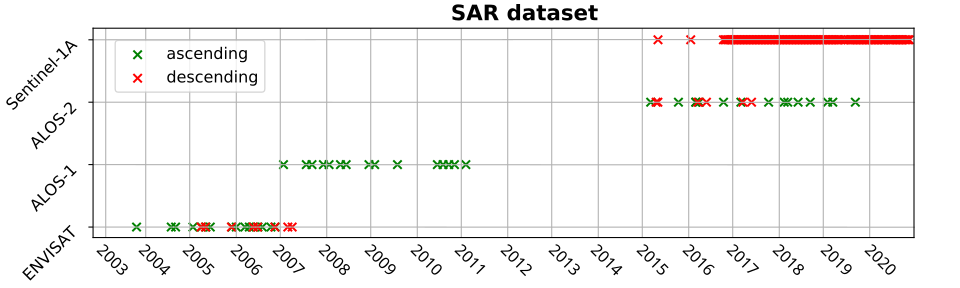


Fig. S1 Time coverage of the SAR data. The horizontal axis shows the acquisition date (years), the vertical axis shows the SAR satellites. Green and red crosses illustrate acquisition timing respectively for ascending and descending orbits. The combination of the four different SAR satellites provides a long time series, whereas the combination of ascending and descending orbits allows analyzing both horizontal and vertical deformation.


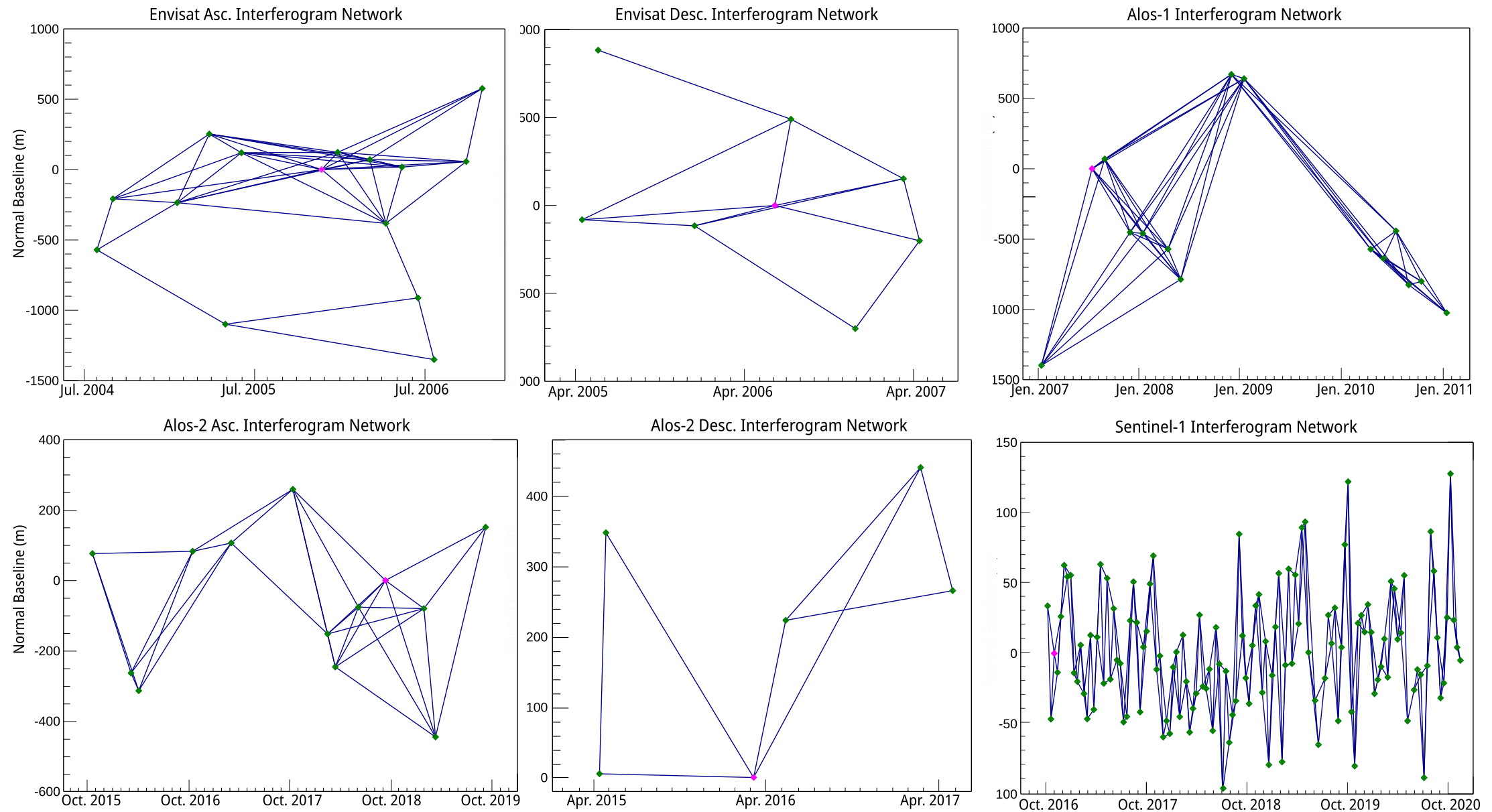
Fig. S2 SBAS connection graphs of the processed SAR dataset, showing the time (x-axis) versus the perpendicular (normal) baseline (y-axis); blue lines indicate the interferograms used for the time series generation


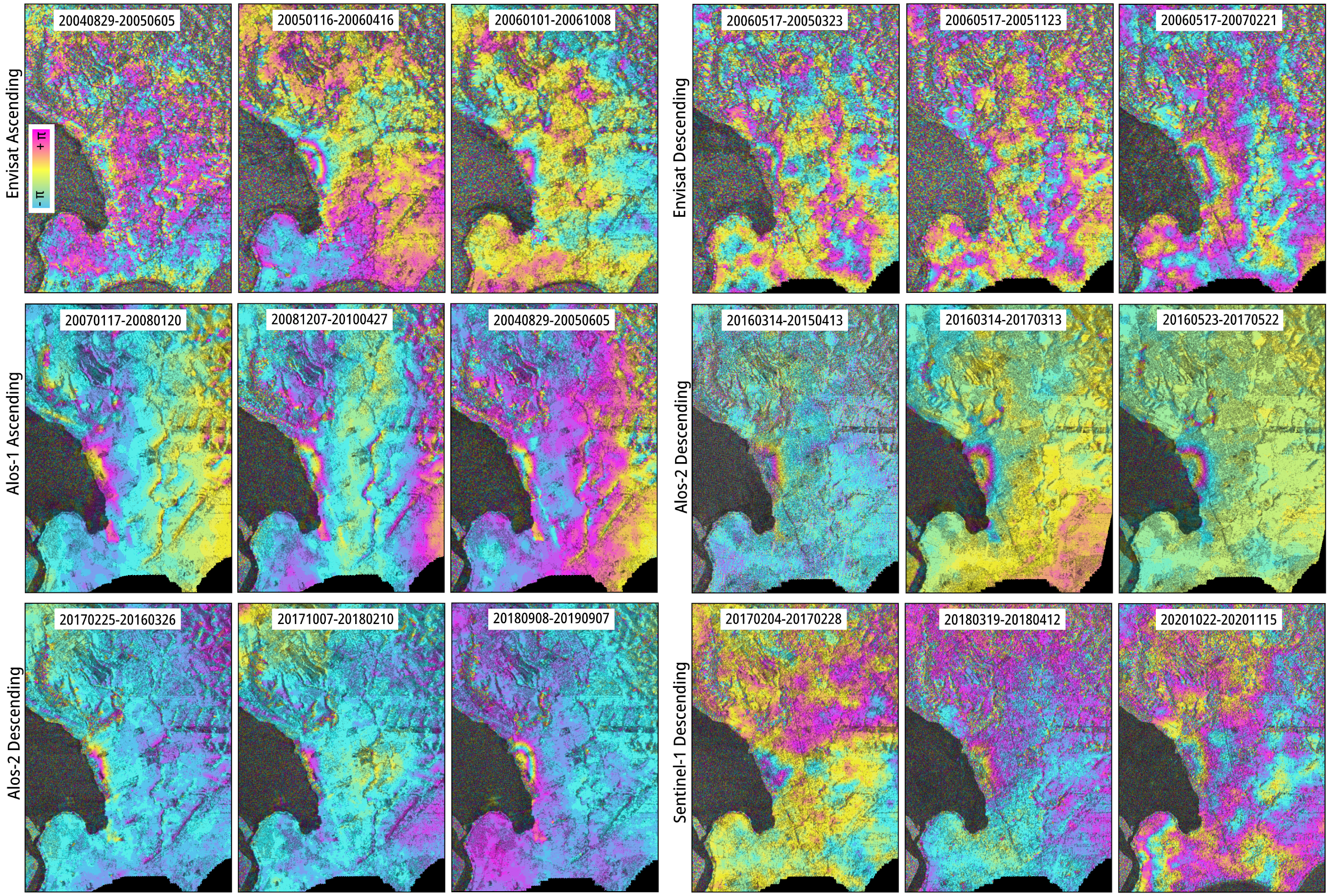


Fig. S3 Selected interferograms (wrapped phase, see legend in upper left image) generated from different sensors; one fringe corresponds to a LOS displacement of approximately 2,8 cm for C-band radar sensors (Envisat and Sentinel-1) and 11.5 cm for L-band sensors (Alos-1 and 2). SAR imagery as background. Water is black. The figures were plotted in QGIS (v. 3.16, https://www.qgis.org/en/site/)


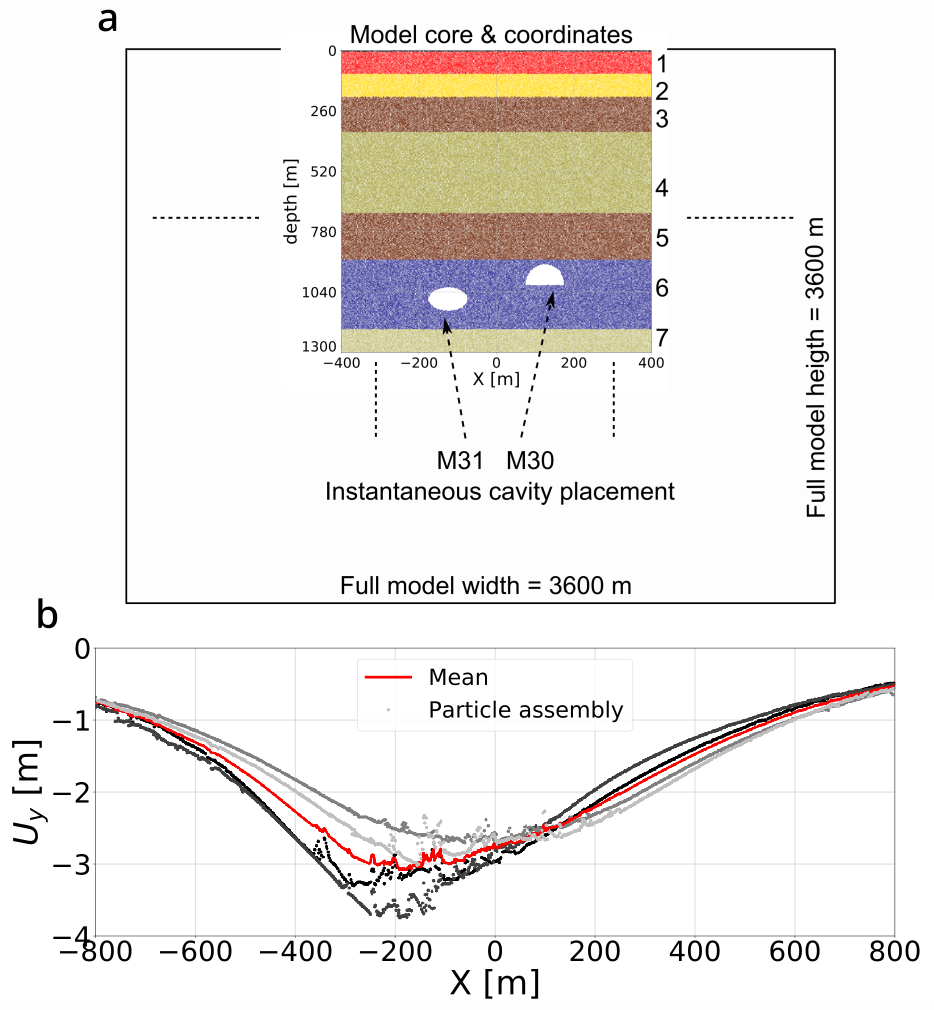


Fig. S4 (**a**) 2D DEM subsidence model setup. The core of the model is shown in the upper center with labeled geologic layers. (**b**) Model uncertainty investigation. The final subsidence (stage 4) along profile P1 (Fig. 3a) for four random particle assemblies of scenario S2 (total cavity collapse) is shown. Red line represents the mean. These plots were assembled using Matplotlib Python library.

| **mission** | **orbit path** | **band** | **Azim. (°)** | **Inc. (°)** | **N.° of images** | **period** | **Vel. error (mm/year)** |
| --- | --- | --- | --- | --- | --- | --- | --- |
| **ASAR ENVISAT** | ASC | C (5.331 GHz) | 24.4 | 76.8 | 15 | 25/07/2004 - 12/11/2006 | 1.8±1.3 |
| **ASAR ENVISAT** | DESC | C (5.331 GHz) | 23.8 | -77.7 | 8 | 23/03/2005 - 28/03/2007 | 2.4±2.5 |
| **ALOS-1 POLSAR** | ASC | L (1.2 GHz) | 37.1 | 78.8 | 16 | 17/01/2007 - 28/01/2011 | -0.8±1.4 |
| **ALOS-2 POLSAR** | DESC | L (1.2 GHz) | 35 | -78.2 | 6 | 13/04/2015 - 22/05/2017 | -1.8±3.0 |
| **ALOS-2 POLSAR** | ASC | L (1.2 GHz) | 35.4 | 77.3 | 13 | 10/10/2015 - 07/09/2019 | 0.2±3.3 |
| **SENTINEL-1A** | DESC | C (5.331 GHz) | 35 | 77.9 | 121 | 07/10/2016 - 15/11/2020 | -1.1±1.8 |

Tab. S1 SAR datasets and main characteristics: Azim. (azimuth angle), Inc. (incidence angle), Vel. error (SBAS average velocity error and error standard deviation).

| **Parameter** | **Symbol** | **Unit** | **(1)**  **Sediments**  **Clay Silt Sand** | **(2)**  **Unconsol. Calcarenite** | **(3)**  **Unconsol. Sandstone Shale** | **(4)**  **Consol. Conglomerates** | **(5)**  **Fract.**  **Sandstone Limestone Shale** | **(6)**  **Salt (halite) Sal-gema** | **(7)**  **Consol.**  **Shale** |
| --- | --- | --- | --- | --- | --- | --- | --- | --- | --- |
| Initial material porosity | n | - | 0.16 | 0.16 | 0.16 | 0.16 | 0.16 | 0.16 | 0.16 |
| Particle density | ρ | kg/m^3^ | 2000 | 2000 | 2000 | 2000 | 2500 | 2170 | 2500 |
| Contact Young’s modulus | E_L_ | GPa | 0.1 | 1 | 1 | 25 | 10 | 3 | 50 |
| Bond Young’s modulus | E_Pb_ | GPa | 0.1 | 1 | 1 | 25 | 10 | 3 | 50 |
| Bond tensile strength | $\underline{\sigma}_{c}$ | MPa | 0.2 | 1 | 1 | 10 | 10 | 14 | 100 |
| Bond cohesion | $\underline{c}$ | MPa | 0.075 | 1 | 1 | 10 | 1 | 1 | 10 |
| Bond friction angle | ϕ | ° | 30 | 30 | 30 | 30 | 30 | 30 | 30 |
| Layer thickness | d | m | 100 | 100 | 150 | 350 | 200 | 300 | 2400 |

Table S2: Material parameters used for the DEM subsidence models. A friction coefficient of 0.5 and a normal/shear stiffness ratio of 2.5 is chosen for all materials.

| **Geometric parameter or Micro-parameter** | **Symbol** | **Unit** | **Value** |
| --- | --- | --- | --- |
| Model height | H | [m] | 3600 |
| Model width | W | [m] | 3600 |
| Minimum particle radius | $R_{min}$ | [m] | 1.25 |
| Mean particle radius | $\underline{R}$ | [m] | 1.66 |
| Solve ratio unbalanced/balanced forces | SR |  | 1^-6^ |
| Boundary walls Young’s modulus | E_W_ | [GPa] | -5 |
| Linear contact friction coefficient | μ |  | 0.01 (initial settling); 0.5 (model) |
| Linear contact normal/shear damping | $\frac{\beta_{n}}{\beta_{s}}$ |  | 0.7/0.0 |
| Surface gap | g_S_ | [m] | 2.1^-2^ * $R_{min}$ (= 1^-4^) |
| Gravitational acceleration | G | [m/s^2^] | 9.81 |
| Cavity M30 centre x/z | x_M30_, h_M30_ | [m] | 125/1010 |
| Cavity M30 long/short axis | a_M30,_ b_M30_ | [m] | 90/50 |
| Cavity M31 centre x/z | x_M31_, h_M31_ | [m] | -125/1070 |
| Cavity M31 long/short axis | a_M31,_ b_M31_ | [m] | 50/50 |

Table S3: Simulation parameters for DEM subsidence models. A friction coefficient of 0.5 and a normal/shear stiffness ratio of 2.5 is chosen for all materials.

| **Hazard class** | **angular distortion** | | **LOS angular distortion** | |
| --- | --- | --- | --- | --- |
| **very low** | < | 0.00033 | < | 0.00027 |
| **low** | 0.00033 | 0.00067 | 0.00027 | 0.00055 |
| **medium** | 0.00067 | 0.002 | 0.00055 | 0.00164 |
| **high** | 0.002 | 0.0067 | 0.00164 | 0.0055 |
| **very high** | 0.0067 | > | 0.0055 | > |

Table S4: Angular distortion thresholds and hazard classification.
